# Unbalanced act: Biased subgenome expression during canola seed development

**DOI:** 10.1093/plphys/kiaf324

**Published:** 2025-07-22

**Authors:** Neeta Lohani

**Affiliations:** Plant Physiology, American Society of Plant Biologists; Department of Biotechnology, Thapar Institute for Engineering and Technology, Patiala, Punjab 147004, India

Understanding how crops coordinate complex developmental programs is essential for maintaining global food security. This challenge becomes particularly difficult in polyploid crops due to the presence of multiple copies of subgenomes (Schaart et al. 2021). Canola (*Brassica napus*), the second most important oilseed crop globally, is an allopolyploid formed approximately 7,500 to 12,500 years ago through interspecific hybridization between *Brassica rapa* and *Brassica oleracea* (Nagaharu and Nagaharu 1935). In canola, the genomes A^r^A^r^ from *B. rapa* and C^o^C^o^ from *B. oleracea* combine to form its complete genome (A^n^A^n^C^n^C^n^) (Chalhoub et al. 2014). These 2 subgenomes must coordinate to regulate the different developmental stages in canola.

Seed development represents a highly intricate coordination challenge, requiring the integration of genetically distinct maternal (seed coat) and filial (embryo and endosperm formed as a result of fertilization) tissues (Li and Yu 2022). In canola, previous studies indicated that gene expression displayed subgenome bias in whole seeds, with transcription factor activity dominated by the A^n^ subgenome (Khan et al. 2022), while DNA methylation and small RNA expression showed bias toward the C^n^ subgenome (Ziegler et al. 2023). However, fundamental questions remain about how this genomic imbalance plays out during the complex process of seed development. Do the 2 subgenomes work as equal partners across all seed tissues, or does genomic “favoritism” vary between maternal and filial compartments? How does subgenome bias change as seed development progresses from fertilization through maturation?

In this issue of *Plant Physiology*, Ziegler et al. (2025) investigated the subgenome bias during seed development in canola by creating a comprehensive spatial and temporal transcriptome atlas. Using laser microdissection coupled with RNA sequencing, the authors isolated 8 distinct seed subregions across 4 developmental stages: ovule, globular (7 days post-pollination), heart (10 days post-pollination), and mature green (28 days post-pollination). From maternal tissues, they captured the inner seed coat, outer seed coat, chalazal seed coat, and chalazal proliferating tissue (CPT) (Figure. A). From filial tissues, they performed sequencing from 3 endosperm compartments (micropylar, peripheral, and chalazal endosperm) and the embryo proper. The mature embryo was further divided into root and cotyledon regions. This comprehensive sequencing approach detected 55,839 genes (78.6% of the annotated genes in *B. napus*), representing a 44% increase in the number of total detected genes compared with the whole-seed transcriptome.

**Figure.**
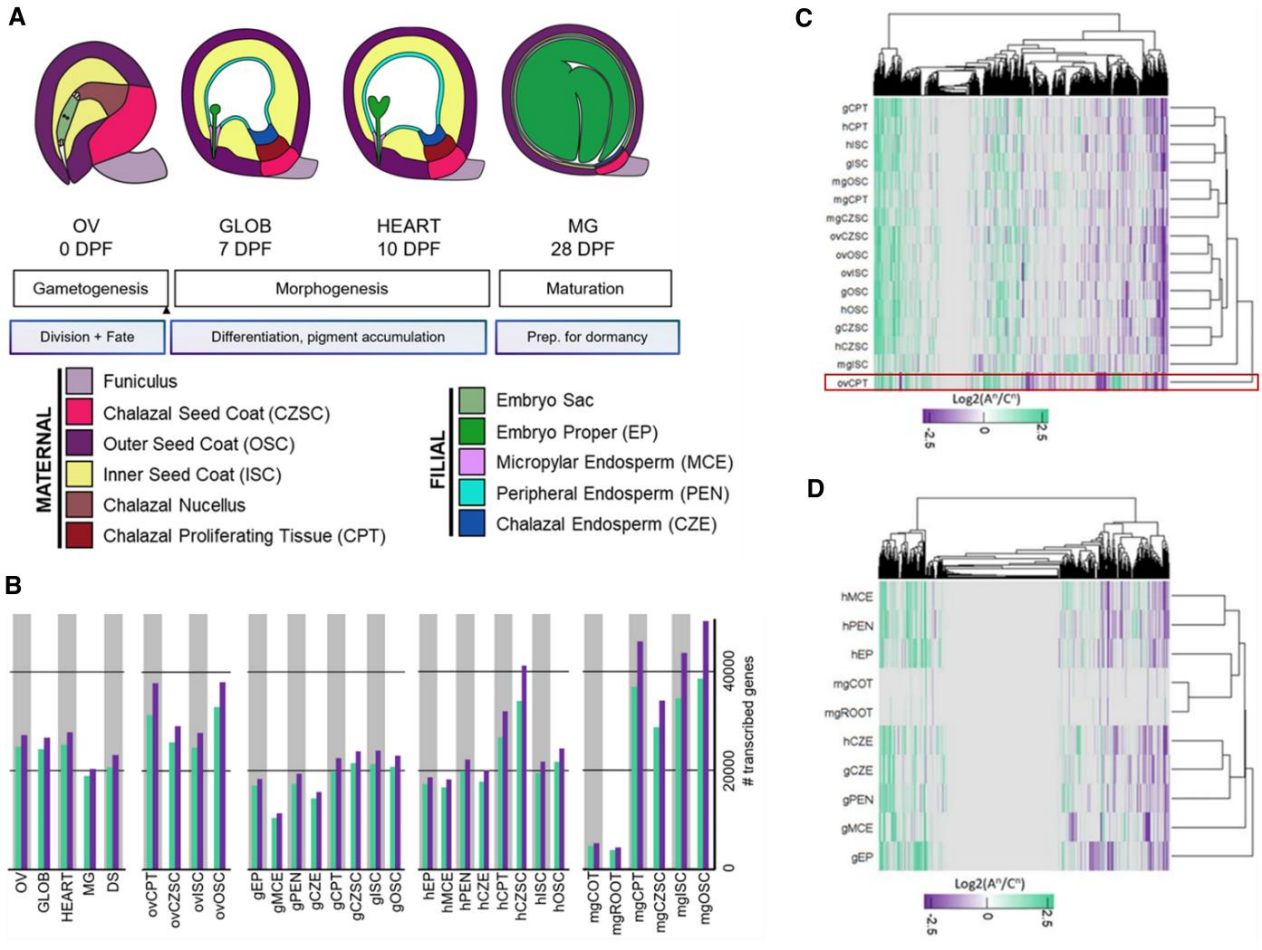
Spatial and temporal transcriptome atlas reveals tissue-specific subgenome bias during canola seed development. **A)** Schematic representation of canola seed development across 4 stages: ovule (OV, 0 days post-pollination, DPP), globular (GLOB, 7 DPP), heart (HEART, 10 DPP), and mature green (MG, 28 DPP). Maternal tissues include seed coat (SC) layers and CPT, while filial tissues comprise embryo and endosperm compartments. **B)** Number of transcribed genes from A^n^ (purple) and C^n^ (green) subgenomes across all seed tissues and developmental stages. **C)** Clustering analysis of homologous gene pairs in maternal seed tissues. Heat map displays Log₂(A^n^/C^n^) expression ratios, where green indicates C^n^ subgenome bias, purple indicates A^n^ subgenome bias, and white represents balanced expression. The ovule CPT (ovCPT, red box) clusters away from all other maternal tissues and shows the most extreme C^n^ bias. **D)** Clustering analysis of homologous gene pairs in filial seed tissues. Filial tissues exhibit more balanced subgenome expressions compared with maternal tissues, with fewer strongly biased gene pairs across development.

The analysis of the RNA-Seq data revealed that the C^n^ subgenome consistently dominates gene expression both in terms of the number of expressed genes and total transcript accumulation across nearly all seed compartments. This bias was most extreme (17.6% A^n^/82% C^n^) in cotyledons from the mature green seed stage and was most balanced (45.4% A^n^/54.5% C^n^) in the CPT from the globular seed stage. Additionally, the most highly expressed genes across all developmental stages were either unbiased or C^n^ subgenome–biased (Figure. B).

The authors performed a clustering analysis of homologous gene pairs and identified the CPT as transcriptionally unique among all maternal regions (Figure. C). The ovule CPT (ovCPT) clusters completely away from all other tissues and developmental stages, showing the highest concentration of strongly C^n^-biased genes across the entire study. The transcriptional programming of CPT shifts dramatically during development. The C^n^-biased genes in ovCPT are enriched for endomembrane systems, vacuole function, and polysaccharide catabolism, while A^n^ subgenome-biased genes are involved in cell growth and hormone transport. During morphogenesis, the globular and heart stage CPT cluster together and share patterns with other maternal tissues (inner seed coat and outer seed coat). The expressed genes in the CPT at these stages are associated with controlling epigenetic maintenance, reproductive transitions, and autophagy. By maturation, the CPT clusters with other mature maternal tissues like the outer seed coat and chalazal seed coat. Thus, CPT's changing transcriptional profile potentially correlates with broader developmental transitions, suggesting this tissue may undergo sequential modifications to adopt new functionality from early morphogenesis to seed maturation.

Key developmental genes regulate the transformation of CPT during seed development. *CYSTEINE ENDOPEPTIDASE 1* (*CEP1*) is expressed predominantly in the CPT and inner seed coat during morphogenesis, likely controlling programmed cell death (Zhang et al. 2014). Anatomically, the CPT originates from the nucellus and undergoes complete reorganization—cells either undergo programmed death or become incorporated into the protective pigment layer. As CPT essentially transforms from reproductive tissue into a protective seed coat, coordinated by dynamic subgenome bias patterns, the results indicate that CPT transformation may represent a critical mechanism for managing complex developmental transitions in polyploid seeds.

Next, the authors report that even within maternal tissues, the inner and outer seed coats showed distinct subgenome bias patterns that reflect their specialized functions. The inner seed coat undergoes cell wall remodeling and protein accumulation during pigment layer formation, while the outer seed coat strengthens its palisade layer and deposits protective mucilage. Genes controlling starch metabolism were predominantly C^n^-biased in both layers, but cell wall modification genes showed complex, layer-specific subgenome expression patterns.

In contrast, filial regions displayed relatively more balanced subgenome expression, though still with an overall C^n^ preference (Figure. D). The globular embryo proper showed the highest proportion of strongly biased gene pairs among filial tissues, while mature cotyledons and roots had only a few strongly biased genes, suggesting that genomic balance emerges as the embryo matures. The endosperm regions revealed that C^n^ subgenome bias correlates with genes involved in cell wall synthesis during cellularization, suggesting that endosperm cellularization may be partially controlled by subgenome-specific expression of cell wall construction machinery.

The authors also explored genes controlling canola's economic value (oil and protein) and observed sophisticated regulation patterns. Lipid biosynthesis genes maintained relatively balanced expression between subgenomes, suggesting this economically vital pathway has been protected from subgenome bias. However, storage protein genes, while showing balanced expression at the gene family level, displayed striking differences between individual gene homologous gene pairs within families. For example, *OLEO1* (oleosin) showed up to 39-fold expression differences between subgenomes, while storage proteins like *SESA4* (seed storage albumin) and *CRU2* (cruciferin) exhibited significant subgenome bias only in specific homologous pairs. This pattern suggests that polyploidization has created opportunities for fine-tuned regulation rather than simple redundancy.

The findings by Ziegler et al. (2025) reveal that the 2 ancestral genomes in canola have evolved specialized roles within specific seed tissues. The previously unexplored CPT emerges as particularly significant for crop development in oilseeds and represents a critical aspect of Brassicaceae seed diversity. The work demonstrates that individual cells, tissues, and organs across the seed lifecycle are influenced by polyploidization in distinct ways while coordinating seed formation. These insights provide new opportunities for developing targeted breeding strategies that leverage specialized subgenome functions in canola. Future functional validation of the identified subgenome-biased genes would enable the application of precise genome editing tools like CRISPR to enhance specific C^n^ subgenome alleles that control seed storage and yield. Additionally, single-cell analysis could provide even finer resolution of these tissue-specific patterns. Similar cell-type resolution studies in other polyploid crops could further reveal whether subgenome coordination mechanisms represent a broader evolutionary principle for crop improvement.

## Data Availability

No new data were generated or analyzed in support of this article.
